# Optimized production of bacterioruberin from “*Haloferax marinum*” using one-factor-at-a-time and central composite design approaches

**DOI:** 10.1186/s40643-024-00834-9

**Published:** 2024-12-19

**Authors:** Eui-Sang Cho, Chi Young Hwang, Myung-Ji Seo

**Affiliations:** 1 BioTechnology Institute, University of Minnesota, St. Paul, MN 55108 USA; 2 Department of Bioengineering and Nano-Bioengineering, Incheon National University, Incheon, 22012 Republic of Korea; 3 Division of Bioengineering, Incheon National University, Incheon, 22012 Republic of Korea; 4 Research Center for Bio Materials and Process Development, Incheon National University, Incheon, 22012 Republic of Korea

**Keywords:** Bacterioruberin, *Haloferax marinum*, Carotenoid, Optimization

## Abstract

Haloarchaea represents a unique group of microorganisms that have adapted to thrive in high-salt environments. These microbes produce distinctive biomolecules, some of which exhibit extraordinary properties. One such biomolecule is bacterioruberin, a prominent red-pigmented C_50_ carotenoid commonly found in halophilic archaea, renowned for its antioxidant properties and potential as a functional resource. This study aimed to enhance the culture conditions for optimal production of C_50_ carotenoids, primarily bacterioruberin, using “*Haloferax marinum*” MBLA0078. The optimization process involved a combination of one-factor-at-a-time (OFAT) and statistical methodology. Under OFAT-optimized conditions, fed-batch fermentation, and response surface methodology (RSM) optimization, carotenoid production reached 0.954 mg/L, 2.80 mg/L, and 2.16 mg/L, respectively, in a 7-L laboratory-scale fermenter. Notably, RSM-optimized conditions led to a 12-fold increase in productivity (0.72 mg/L/day) compared to the basal DBCM2 medium (0.06 mg/L/day). These findings suggest that strain MBLA0078 holds significant promise for commercial-scale production of bacterioruberin.

**Graphical Abstract:**

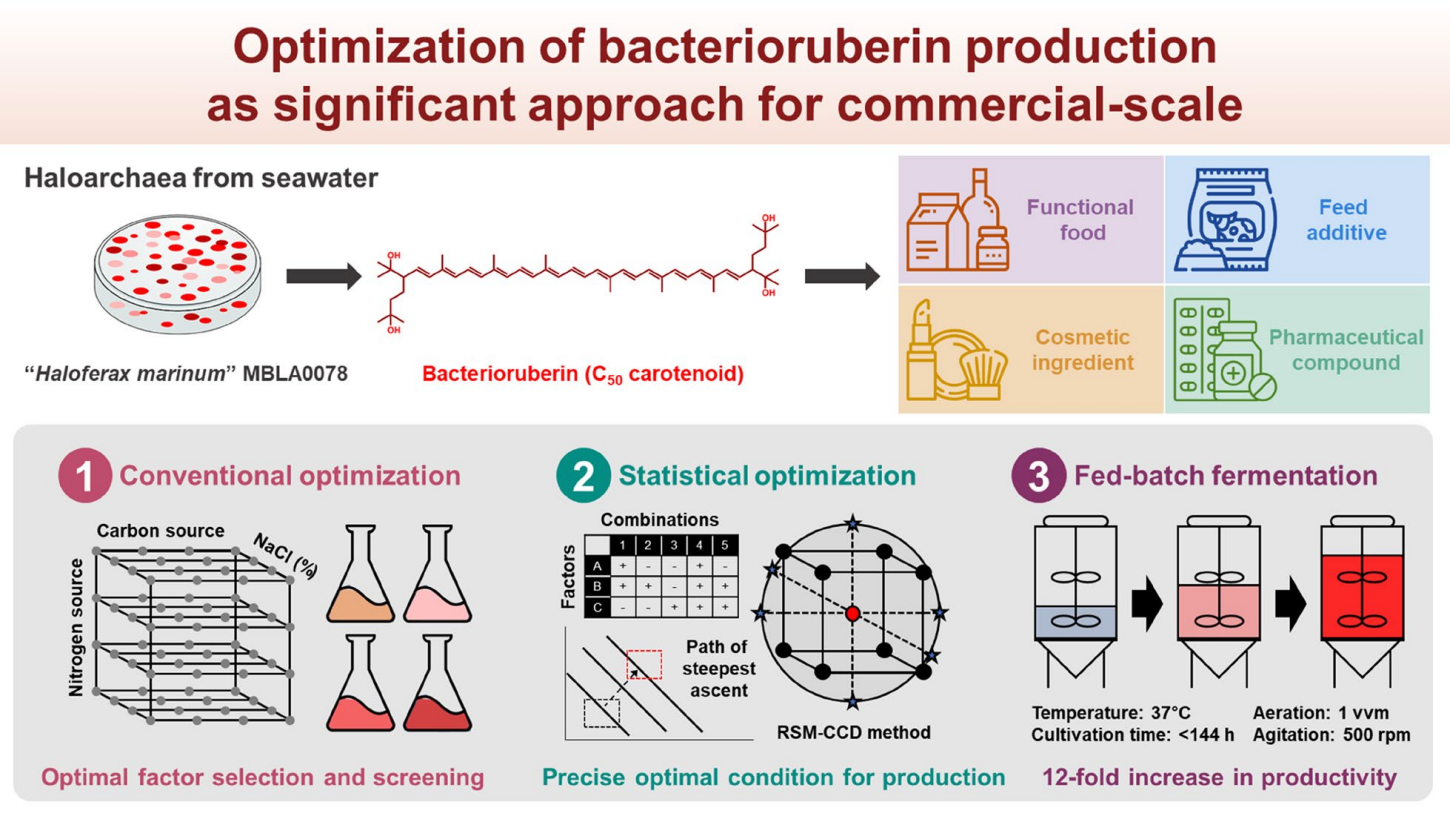

**Supplementary Information:**

The online version contains supplementary material available at 10.1186/s40643-024-00834-9.

## Introduction

Carotenoids, also known as tetraterpenoids, are natural pigments found in a wide range of plants, algae, and microorganisms (Alcaino et al. 2016). As a subfamily of lipophilic isoprenoids, carotenoids are among the most ubiquitous natural compounds, displaying a spectrum of colors from yellow to red (Borowitzka 2010). These pigments are derived from the terpene biosynthetic pathway and are categorized by the length of their carbon backbones (C_30_, C_40_, and C_50_) (Heider et al. 2014). Carotenoids have attracted significant interest in the food industry for their role as natural colorants and are increasingly acknowledged as valuable functional bio-resources due to their strong antioxidant and anticancer properties. In addition, their potential applications are being explored in the pharmaceutical and cosmeceutical fields (Squillaci et al. 2017).

Currently, concerns about the health implications of synthetic carotenoids have led to an increasing demand for natural carotenoids derived from microorganisms (Celedón and Díaz 2021). Microbial cultures allow for precise control over environmental conditions, such as temperature, pH, and nutrient levels, to enhance carotenoid yield (Paliwal et al. 2017; Ram et al. 2019). Microbial platforms are flexible, readily engineered to synthesize a wide range of carotenoids, and can use renewable resources like sugars and agricultural by-products, making them economically and environmentally sustainable (Ram et al. 2020). Many microorganisms have been explored as potential carotenoid production platforms, with microalgae being the most extensively studied (Forján et al. 2015). Microorganisms, such as the microalgae *Dunaliella salina* and *Haematococcus pluvialis*, are widely used to produce β-carotene and astaxanthin, respectively, while fungi like *Blakeslea trispora* produce lycopene and β-carotene​ (Borowitzka 2010; Berman et al. 2015). However, the potential of haloarchaea as carotenoid producers has been largely underappreciated, despite their ability to synthesize and accumulate both C_40_ and C_50_ carotenoids (de la Vega et al. 2016; Squillaci et al. 2017; Montero-Lobato et al. 2018). Haloarchaea thrive in high-salt environments, which minimizes contamination risks and reduces the need for sterilization. Furthermore, these organisms can also utilize cost-effective substrates such as seawater and waste streams (Oren 2010). Although their biomass productivity is currently low, optimization of their cultivation systems and extraction methods could increase their economic viability and carotenoid yield, potentially surpassing that of traditional microalgae (Fang et al. 2010). Several haloarchaea species have been reported to accumulate carotenoids intracellularly at levels up to 0–25 mg/g (2–2.5% of biomass dry weight), even higher than most microalgae (Hamidi et al. 2014; Xu et al. 2018).

Bacterioruberin (BR) is a primary C_50_ carotenoid produced by haloarchaea, with promising applications in the food, cosmetic, and pharmaceutical industries due to its potent antioxidant properties (Squillaci et al. 2017). “*Haloferax marinum*” strain MBLA0078, a halophilic archaeon isolated from seawater, is another notable producer of BR and its precursors (Cho et al. 2021). BR production in haloarchaea is known to be strongly influenced by NaCl concentration, which suggests that “*Hfx. marinum*”, capable of thriving in a wide range of salinity conditions, could be a promising candidate for BR production (Fang et al. 2010; Chen et al. 2015; Cho et al. 2021). Moreover, BR extracts derived from “*Hfx. marinum*” have demonstrated significant antioxidant activity and protective effects against muscle atrophy, highlighting the potential therapeutic applications of this microorganism (Lee et al. 2024).

Thus, this study aims to optimize the culture conditions for carotenoid production using one-factor-at-a-time (OFAT) and statistical approaches, including Plackett-Burman (PB) and response surface methodology (RSM). These optimized conditions resulted in increased bacterioruberin production compared to the basal medium, with cultivation in a lab-scale fermenter further enhancing BR productivity.

## Materials and methods

### Strain and culture medium

“*Haloferax marinum*” MBLA0078 (= KCTC 4290 = JCM 34171) was previously isolated from seawater collected near Yeoungheungdo Island in the Yellow Sea, Republic of Korea (Cho et al. 2021). The strain was cultured in DB Characterization Medium No. 2 (DBCM2), which contains the following components per liter: 833 mL MDS salt water (composed of 5 g KCl, 35 g MgSO_4_·7H_2_O, 30 g MgCl_2_·6H_2_O, 240 g NaCl, and 5 mL 1 M CaCl_2_ solution), 1 mL of 0.15% (w/v) FeCl_2_·4H_2_O solution, 1 mL of trace element solution (0.10 g ZnSO_4_·7H_2_O, 0.03 g MnCl_2_·4H_2_O, 0.3 g H_3_BO_3_, 0.2 g CoCl_2_·6H_2_O, 0.01 g CuCl_2_·2H_2_O, 0.02 g NiCl_2_·6H_2_O, and 0.03 g Na_2_MoO_4_·2H_2_O), 0.25 g of peptone (Oxoid), 0.05 g of yeast extract (Difco), 5 mL of 1 M NH_4_Cl, 2 mL of 0.5 M potassium phosphate buffer, 3 mL of vitamin solution (3.0 mg biotin, 4.0 mg folic acid, 50.0 mg pyridoxine·HCl, 33.0 mg thiamine·HCl, 10 mg riboflavin, 33.0 mg nicotinic acid, 17 mg DL-calcium pantothenate, 17 mg vitamin B12, 13 mg p-aminobenzoic acid, and 10 mg lipoic acid), and 10 mL of 1 M sodium pyruvate solution (Burns et al. 2010). The pH was adjusted to pH 7.5. The seed culture was prepared by inoculating 20 mL of liquid medium into a 50 mL conical tube and incubating it overnight at 37 °C with agitation at 180 rpm. Unless otherwise specified, these cultures were used as inoculum at a concentration of 1% (v/v) for the main culture. The cultures were grown under standard aerobic conditions in 250-mL Erlenmeyer flasks (working volume: 100 mL) at 180 rpm and 37 °C for 3 days to achieve maximum carotenoid production. Throughout the optimization process, all non-variables components of the medium were prepared according to the DBCM2 formulation.

### Extraction and quantification of carotenoids

Following the cultivation, the culture broth was centrifuged at 10,000 rpm for 5 min to harvest the cells. The cell pellet was then suspended in a 7:3 (v/v) acetone: methanol solution and stirred until the pellet turned white, indicating complete pigment extraction. This suspension was centrifuged again at 13,000 rpm for 10 min, and the supernatant was collected. The supernatant was evaporated, diluted to a constant volume with methanol, and the absorbance was measured at 494 nm. The total carotenoid content was quantified by measuring the absorbance at 494 nm and calculated using an extinction coefficient, ε of 2660 (1%) and calculated using the following formula: carotenoid production (mg/L) = (A_494_/2660) x 10^4^ (Mandelli et al. 2012; Hwang et al. 2024).

### Optimization by classical OFAT method

Carotenoid production by “*Hfx. marinum*” MBLA0078 was optimized using an OFAT approach, testing various carbon and nitrogen sources. Different carbon sources (0.1% w/v), including glucose, galactose, fructose, maltose, sucrose, lactose, starch, and glycerol, were evaluated. Glucose concentrations ranging from 0.10 to 1.50% (w/v) were tested for further optimization. For nitrogen source selection, yeast extract and peptone were replaced with one of the following: peptone, fish peptone, yeast extract, skim milk, gelatin, casamino acids, soytone, tryptone, or beef extract, each at 1 g/L. Fish peptone concentrations were optimized by varying between 0.05 and 0.50% (w/v). Sodium chloride concentrations were also optimized, ranging from 3 to 30% (w/v). The final optimized medium, developed using the OFAT method, was designated as DBCM2O medium.

### Screening of significant variables

The PB design was performed to screen the significant variables (factors) that influenced carotenoid production. Ten variables of medium components and fermentation conditions were tested at low (–1) and high (+ 1) levels, including the carbon and nitrogen sources selected through the OFAT method, along with NH_4_Cl, NaCl, MgSO_4_·7H_2_O, MgCl_2_·6H_2_O, KCl, and CaCl_2_·2H_2_O. Additionally, inoculum size (IS) and incubation time (IT) were selected as critical experimental factors, given their significant impact on production and fermentation process. The levels of each variable are provided in Table 1. A 12-run experiment was generated using Minitab software (Minitab 18 Trial, Minitab Inc., USA) (Table 2). The experimental design followed the PB method, based on the first-order model:

**Table 1:** Range of variables of the Plackett-Burman design

Symbol code	Variables	Units	Experimental values		
			Low (-1)	Center (0)	High (+ 1)
X_1_	Glucose	g/L	1	5.5	10
X_2_	Fish peptone	g/L	1	5.5	10
X_3_	1 M NH_4_Cl solution	mL	5	7.5	10
X_4_	NaCl	g/L	70	160	250
X_5_	MgSO_4_∙7H_2_O	g/L	30	45	60
X_6_	MgCl_2_∙6H_2_O	g/L	25	33.5	50
X_7_	KCl	g/L	6	9	12
X_8_	1 M CaCl_2_ solution	mL	4	6	8
X_9_	Inoculum volume (IV)	% (v/v)	5	7.5	10
X_10_	Incubation time (IT)	H	48	72	96

**Table 2:** Plackett–Burman experimental design for screening of the medium components and fermentation conditions that affected the actual and predicted carotenoid production (mg/L). X_1_, Glucose; X_2_, Fish peptone; X_3_, 1 M NH_4_Cl solution; X_4_, NaCl; X_5_, MgSO_4_∙7H_2_O; X_6_, MgCl_2_∙6H_2_O; X_7_, KCl; X_8_, 1 M CaCl_2_ solution; X_9_, Inoculum volume; X_10_, Incubation time

STD order	Coded variable level											Carotenoid production (mg/L)		
	X_1_	X_2_	X_3_	X_4_	X_5_	X_6_	X_7_	X_8_	X_9_	X_10_	Actual		Predicted	
1	1	-1	1	-1	-1	-1	1	1	1	-1	0.51		0.53	
2	1	1	-1	1	-1	-1	-1	1	1	1	0		-0.02	
3	-1	1	1	-1	1	-1	-1	-1	1	1	0.63		0.65	
4	1	-1	1	1	-1	1	-1	-1	-1	1	0.16		0.18	
5	1	1	-1	1	1	-1	1	-1	-1	-1	0		0.02	
6	1	1	1	-1	1	1	-1	1	-1	-1	0.27		0.25	
7	-1	1	1	1	-1	1	1	-1	1	-1	0		-0.02	
8	-1	-1	1	1	1	-1	1	1	-1	1	0.51		0.49	
9	-1	-1	-1	1	1	1	-1	1	1	-1	0.12		0.14	
10	1	-1	-1	-1	1	1	1	-1	1	1	1.18		1.16	
11	-1	1	-1	-1	-1	1	1	1	-1	1	0.86		0.88	
12	-1	-1	-1	-1	-1	-1	-1	-1	-1	-1	0.51		0.49	
13	0	0	0	0	0	0	0	0	0	0	1.18		1.14	
14	0	0	0	0	0	0	0	0	0	0	1.18		1.14	
15	0	0	0	0	0	0	0	0	0	0	1.06		1.14	

$$\:Y={\beta\:}_{0}+{\sum\:}_{i=1}^{k}{\beta\:}_{i}{x}_{i}$$

where *Y* represents carotenoid production, *β*_*0*_ is the model intercept, *β*_*i*_ denotes the linear coefficient, *x*_*i*_ is the level of the independent variable, and *k* is the number of variables. All experiments were performed in triplicate, and the average carotenoid production from each trial was used as the response variable. Regression analysis identified the variables with a significant effect (at a 95% confidence level) on carotenoid production, which were then further optimized in the procedure.

### Path of steepest ascent (or descent) design

Given that initial experimental conditions are often far from optimal, a method is required to efficiently approach the optimum vicinity. The steepest ascent (or descent) method is an effective experimental technique used to move towards the maximum response (Guo et al. 2009). In the first-order model from PB design, the response surface contours consist of parallel lines (Liu et al. 2011). The significant variables identified in the PB design were further optimized to determine the path of maximal improvement. The expected path of the steepest ascent (or descent) was determined by slope Y, which is perpendicular to the contours of the response surface. The step sizes along the path were proportional to the coefficient (*β*_*i*_). This path starts from the center (zero level) of the variables in the PB design and continues until no further increase in the response is observed. The experimental design of the steepest ascent (or descent) method is provided in Table 3.

**Table 3:** Experimental design and response value of path of steepest ascent (decent)

Run	Fish peptone (g/L)	NaCl (g/L)	KCl (g/L)	IT (Hour)	Carotenoid production (mg/L)
Base point ^a^	5.5	160	9	72	1.14
Origin step unit ^b^	4.5	90	3	24	
Slope ^c^	-0.1	-0.26	0.11	0.16	
Proportion ^d^	-0.45	-23.4	0.33	3.84	
New step unit with a decimal	-0.5	-23.0	0.3	4	
Experiment 1	5	137	9.3	76	1.33
Experiment 2	4.5	114	9.6	80	0.95
Experiment 3	4	91	9.9	84	0.79
Experiment 4	3.5	68	10.2	88	0.59
Experiment 5	3	45	10.5	92	0.15

^a^ Zero level in the PB design

^b^ Range of the unity level

^c^ Estimated coefficient ratio from Eq

^d^ Origin step unit X slope

### Central composite design

To determine the optimal conditions for carotenoid production, including fish peptone, NaCl, KCl, and incubation time, a central composite design (CCD) with five coded levels was employed. The four significant variables were tested at five coded levels (–2, − 1, 0, + 1, +2), as shown in Table 4. The center point was set based on the values determined by the results following the path of steepest ascent (or descent) design. The CCD encompassed 30 experimental trials (= 2^k^ + 2k + 6, where k is the number of variables) including 16 factorial design trials, 8 axial point trials (2 per each variable) and 6 replicates of the central point (Chuprom et al. 2016) (Table 5). Other media components and fermentation conditions were set at the center-level concentrations from the PB design (*P* > 0.05). The CCD results were described using the following second-order polynomial derived through multiple regression analysis:

**Table 4:** Levels of the factors chosen for the central composite design experimental design

Symbol code	Variables	Units	Experimental values				
			-2	-1	0	1	2
A	Fish peptone	g/L	1	3	5	7	9
B	NaCl	g/L	25	80	135	190	245
C	KCl	g/L	0	5	10	15	20
D	Incubation Time	h	24	48	72	96	120

**Table 5:** Central composite design matrix of independent variables for the experimental design along with actual and predicted responses for carotenoid production (mg/L)

Std	A	B	C	D	Carotenoid production (mg/L)	
					Actual	Predicted
1	-1	-1	-1	-1	0.71	0.56
2	1	-1	-1	-1	0.93	0.91
3	-1	1	-1	-1	0.52	0.53
4	1	1	-1	-1	0.62	0.47
5	-1	-1	1	-1	0.81	0.82
6	1	-1	1	-1	1.51	1.34
7	-1	1	1	-1	0.67	0.58
8	1	1	1	-1	0.61	0.69
9	-1	-1	-1	1	0.59	0.49
10	1	-1	-1	1	0.94	0.92
11	-1	1	-1	1	0.49	0.55
12	1	1	-1	1	0.60	0.58
13	-1	-1	1	1	0.73	0.77
14	1	-1	1	1	1.41	1.38
15	-1	1	1	1	0.61	0.62
16	1	1	1	1	0.79	0.83
17	-2	0	0	0	0.60	0.65
18	2	0	0	0	1.12	1.19
19	0	-2	0	0	0.60	0.76
20	0	2	0	0	0.21	0.17
21	0	0	-2	0	0.63	0.76
22	0	0	2	0	1.27	1.26
23	0	0	0	-2	0.41	0.59
24	0	0	0	2	0.70	0.65
25	0	0	0	0	1.20	1.32
26	0	0	0	0	1.36	1.32
27	0	0	0	0	1.29	1.32
28	0	0	0	0	1.32	1.32
29	0	0	0	0	1.36	1.32
30	0	0	0	0	1.39	1.32

$$\:Y={\beta\:}_{0}+\sum\:{\beta\:}_{i}{x}_{i}+\sum\:{\beta\:}_{ii}{x}_{i}^{2}+\:\sum\:{\beta\:}_{ij}{x}_{i}{x}_{j}\:\:$$

where *Y* represents the predicted response, *β*_*0*_ is the intercept term, *β*_*i*_ are the linear coefficients, *β*_*ii*_ are the quadratic coefficients, *β*_*ij*_ are the interaction coefficients, and *x*_*i*_ and *x*_*j*_ represent the coded independent variables. To validate the response surface model, experiments were conducted using the conditions predicted by the model, and the results were compared with the predicted values. The “R” programming language was used to generate the response surface plots. Statistical analysis, including ANOVA, was performed to assess the model, while Turkey’s multiple range test was used for mean comparison. The best medium conditions obtained through RSM optimization were designated as the DBCM2R medium.

### Scale up fermentation for carotenoid production

Bacterioruberin production by “*Hfx. marinum*” strain MBLA0078 was conducted in a 7 L laboratory fermenter equipped with a six-blade turbine agitator (Kobiotech; Republic of Korea). The working volume was set to 4.2 L using the production media DBCM2O and DBCM2R, respectively. Before fermentation, the optimized medium was autoclaved at 121 °C for 30 min, followed by inoculation with a 7.5% (v/v) inoculum. Fermentation was carried out at 37 °C with an agitation speed of 500 rpm and an aeration rate of 1.0 vvm. Foaming was controlled with the addition of commercial Antifoam 204 (Sigma, USA). For carotenoid production using DBCM2O, feeding commenced at 48 h when the glucose concentration reached zero, with glucose supplemented to a concentration of 5 g/L. Cultivations was continued for appropriate durations, with samples collected every 12 h for the first 2 days and every 24 h thereafter. The biomass pellet was collected for carotenoid extraction and cell growth analysis.

## Results and discussion

### Optimization of carotenoid production by OFAT approach

**Fig. 1:**
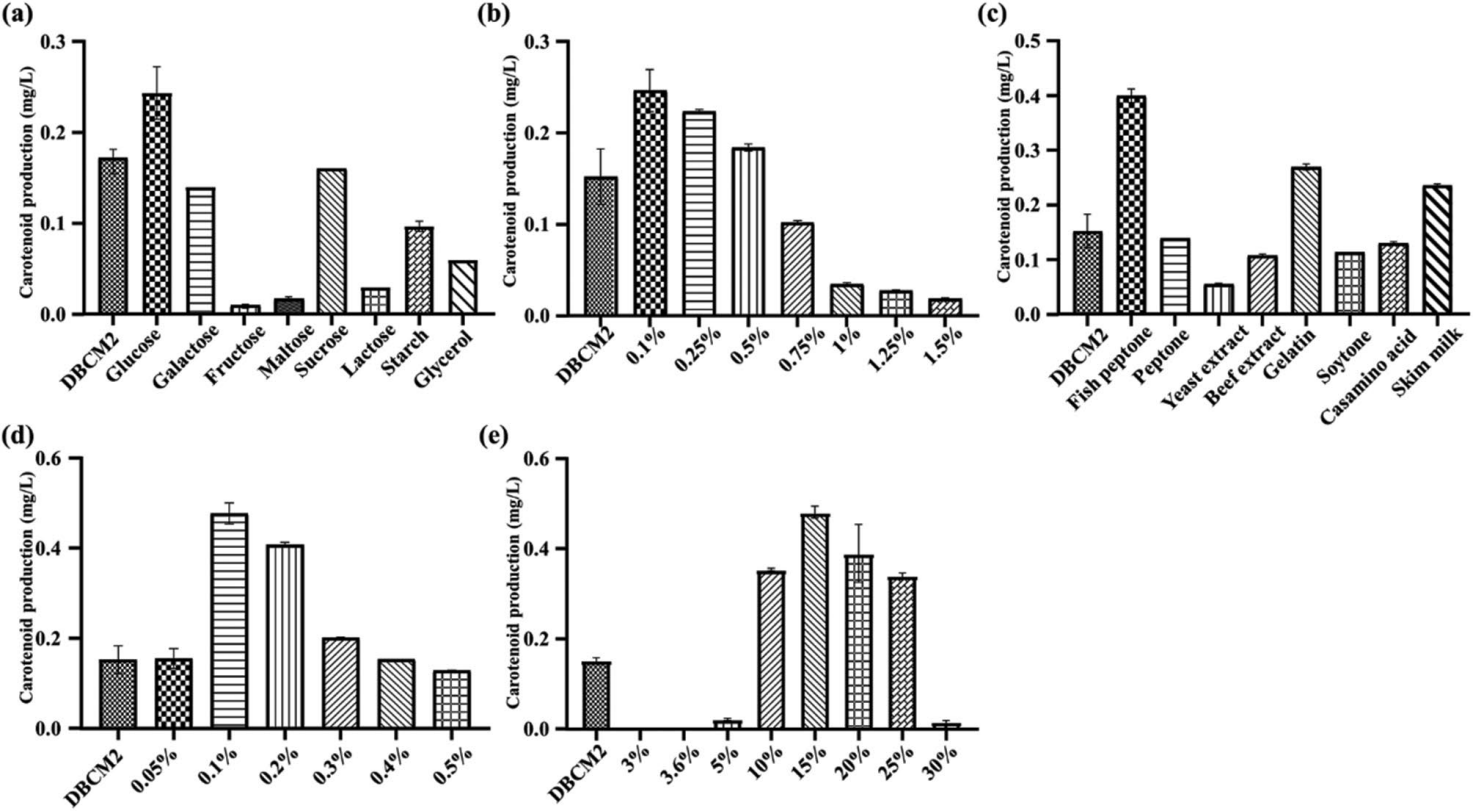
Cho et al. Optimization of carotenoid production by “*Hfx. marinum*” MBLA0078 by OFAT approach. Effect of (**a**) different carbon source [0.1% (w/v)], (**b**) glucose concentration [% (w/v)], (**c**) different nitrogen source [0.1% (w/v)], (**d**) fish peptone concentration [% (w/v)], and (**e**) different NaCl concentration [% (w/v)] on carotenoid production under flask culture. The parameter optimized was incorporated in subsequent experiment

Carotenoid production by “*Hfx. marinum*” MBLA0078 was assessed using various carbon sources, including glucose, galactose, fructose, maltose, sucrose, lactose, starch, and glycerol (each at 0.1% w/v) in a carotenoid production medium, incubated at 37 °C for 72 h. As shown in Fig. 1a, glucose showed the highest carotenoid production at 0.253 mg/L, outperforming the original DBCM2 medium (0.167 mg/L) and other carbon sources, such as sucrose (0.166 mg/L) and galactose (0.149 mg/L). Further, investigation into the effect of glucose concentration revealed that the highest carotenoid production (0.271 mg/L) occurred at 0.1% (w/v) glucose (Fig. 1b). For nitrogen source optimization, “*Hfx. marinum*” MBLA0078 was cultured in DBCM2 medium without yeast extract and peptone, supplemented with 0.1% (w/v) of different nitrogen sources, including peptone, fish peptone, yeast extract, skim milk, gelatin, casamino acids, soytone, tryptone, and beef extract. Among these, fish peptone exhibited the highest carotenoid production at 0.417 mg/L (Fig. 1c). The optimal concentration of fish peptone for carotenoid production by strain MBLA0078 was determined by testing concentrations of 0.05%, 0.1%, 0.2%, 0.3%, 0.4%, and 0.5% (w/v). The maximum carotenoid production (0.477 mg/L) was obtained at 0.1% (w/v) fish peptone (Fig. 1d). To further optimize NaCl concentration, “*Hfx. marinum*” MBLA0078 was inoculated in the DBCM2 medium optimized with the best-performing carbon and nitrogen sources and incubated with varying NaCl concentrations. The highest carotenoid production (0.501 mg/L) was achieved at 15% (w/v) NaCl (Fig. 1e).

### Screening of significant variables using PB experimental design for statistical optimization

To facilitate statistical optimization, a PB design was employed to screen and evaluate the significant variables affecting carotenoid production. Fifteen experimental runs were conducted to analyze the influence of 10 different variables. The design matrix used for screening significant factors, along with the corresponding carotenoid production responses, is presented in Table 2. Carotenoid production ranged widely, from 0 to 1.18 mg/L. The ANOVA results for the PB design related to carotenoid production are provided in Table S1. The coefficient of determination (R^2^) for the first-order model was 0.996, indicating that the model effectively explained the variability in the data. The following first-order polynomial model was derived through regression analysis:

$$\eqalign{ Carotenoid production \left({mg/L} \right) = & 0.110 - 0.00944 {X_1} \cr & - 0.02278 {X_2} - 0.01967 {X_3} \cr & - 0.002935 {X_4} + 0.00372 {X_5} + 0.00287 {X_6} \cr & + 0.003806 {X_7} - 0.00875 {X_8} \cr & + 0.00433 {X_9} + 0.006701 {X_{10}} \cr}$$

where X_1_ = glucose, X_2_ = fish peptone, X_3_ = NH_4_Cl, X_4_ = NaCl, X_5_ = MgSO_4_·7H_2_O, X_6_ = MgCl_2_·6H_2_O, X_7_ = KCl, X_8_ = CaCl_2_·2H_2_O, X_9_ = inoculum volume, X_10_ = incubation time.

Generally, a model term is considered significant when its “*P*-value” is less than 0.05. In this study, fish peptone, NaCl, KCl, and incubation time emerged as significant factors. According to the PB design, reducing NaCl from a high level (25%) to a low level (7%), significantly improved carotenoid production. In the OFAT optimization process, carotenoid production also increased when the NaCl concentration was lowered from 20 to 15% (w/v) (Fig. 1e). Similar results have been reported in response to osmotic stress induced by NaCl concentrations below 20% for *Hfx. mediterranei* ATCC 33,500 (Fang et al. 2010; Chen et al. 2015). Conversely, increasing KCl from a low level (0.6%) to a high level (1.2%) enhanced carotenoid production. Potassium ions appear to be critical for carotenoid production, as they are required in high concentrations for the stability of hydrophilic proteins and the activity of specific enzymes (Kumar and Tiwari 2019). In the OFAT optimization, glucose was identified as the optimal carbon source for carotenoid production, consistent with previous studies on *Hfx. mediterranei*, *Haloarcula* sp. M1, *Halorubrum* sp. M5, and *Halorubrum* sp. HRM-150 (Vázquez-Madrigal et al. 2021; Giani et al. 2022; Ma et al. 2024). However, fish peptone was found to be a more influential factor for carotenoid production than glucose (Table S1). This is likely due to the preference of most haloarchaea, including strain MBLA0078, for organic nitrogen sources, as they require amino acids for growth and energy metabolism (Esclapez et al. 2016; Hwang et al. 2024). Consequently, complex nitrogen sources are anticipated to be favored by strain MBLA0078 for cell growth and secondary metabolite production, pattern observed in other haloarchaea (Giani et al. 2021; Hwang et al. 2024). Therefore, fish peptone, NaCl, KCl, and incubation time were selected as the most critical variables for further optimization using the path of steepest ascent (or descent) and CCD.

### Path of steepest ascent (descent) experiment

The steepest ascent (or descent) method is a sequential procedure that progresses along the path of the most rapid response change. This experiment began at the center of the PB design and proceeded along the path where fish peptone and NaCl concentrations decreased, while KCl concentration and incubation time increased. The step sizes along this path were proportional to the regression coefficients *β*_*i*_. Table 3 indicates the experimental design for the steepest ascent (descent) and the corresponding responses. The highest response (1.33 mg/L) was observed at the first step, with the following medium conditions: 5 g/L fish peptone, 137 g/L NaCl, 9.3 g/L KCl, and 76 h of incubation time (Table 3). This point was found to be near the region of maximum carotenoid production without considering interactions between the factors. As result, the optimal center point for subsequent optimization was chosen: 5 g/L fish peptone, 135 g/L NaCl, 10 g/L KCl and 72 h incubation time.

### Optimization of carotenoid production using CCD based RSM

Carotenoid production by “*Hfx. marinum*” MBLA0078 was optimized using CCD based RSM, exploring a range of four significant variables: fish peptone (A), NaCl (B), KCl (C), and incubation time (D). All the other factors for carotenoid production were maintained at their median levels as they obtained in preliminary PB design. A total of 30 experimental runs were performed, varying the combination of these four variables. The design matrix and the corresponding experimental results, used to assess the effects of the independent variables, are presented in Table 6. Coefficients and *P*-values for all linear (A, B, C, D), quadratic (A^2^, B^2^, C^2^, D^2^) and interaction (AB, AC, AD, BC, BD, CD) terms were calculated and shown in Table S2. Among the linear terms, A, B, and C had significant effects on carotenoid production (*P* < 0.05). For the quadratic terms, A^2^, B^2^, C^2^, and D^2^ were also significant (*P* < 0.05). However, only the AB interaction term was significant (*P* < 0.05), while the other interaction terms (AC, BC, BD, CD) were not (*P* > 0.05). A second-order polynomial quadratic regression equation for carotenoid production was developed based on the experimental results, as follows:

**Table 6:** Optimal parameter estimates for lab scale fermentation kinetic models

Condition	Total carotenoid production			Cell growth			Y_*P*/X_^d^ (µg/g)
	Carotenoid production (mg/L)	Y_*P*/S_^a^ (mg/g)	Productivity (mg/L/day)	Biomass production (g/L)	Y_X/S_^b^ (g/g)	µ^c^ (h^− 1^)	
DBCMO	0.95	0.95	0.32	1.25	1.25	0.0772	760.00
Fed-batch fermentation	2.8	0.56	0.47	4.37	0.874	0.0371	640.73
DBCMR	2.16	1.02	0.72	2.21	1.05	0.1187	977.38

^a^ Product yield (carotenoid production mg / glucose g)

^b^ Biomass yield (Dry cell weight g / glucose g)

^c^ Specific growth rate

^d^ Product yield coefficient (carotenoid production µg / Biomass g)

$$\eqalign{ Carotenoid production \left({mg/L} \right) & = \cr & -5.436 + 0.428 A + 0.05210 B \cr & + 0.1460 C + 0.03801 D \cr & - 0.02509 {A^2} - 0.000175 {B^2} \cr & - 0.00483 {C^2}- 0.000306 {D^2} \cr & - 0.001465 AB + 0.00562 AC \cr & + 0.000457 AD- 0.0000374 BC \cr & + 0.000028 BD + 0.000063 CD \cr}$$

The model’s fit was indicated by the coefficient of determination (R^2^ = 0.942), showing that 94.2% of the variability in the response could be explained by the model (Table S2). Statistical significance of the second-order model was confirmed by ANOVA through the *F*-test. The “Model *F*-value” was highly significant, with only a 0.01% likelihood of being influenced by noise (*P* < 0.0001) (Table S2). In addition, non-significant lack of fit (0.07) confirmed the validity of the quadratic model in this study. The coefficient of variation (CV), which represents the ratio of the standard error to the mean value, was low (0.12%), indicating good reproducibility of the experiment, as models with CVs below 10% are generally considered reliable (Liu et al. 2011).

Three-dimensional (3D) response surface contour plots were generated to illustrate the relationships between the response and the experimental levels of each variable (Fig. S1). Each plot shows the effect of two variables while the other factors were held constant at their zero levels. For fish peptone and NaCl, carotenoid production reached optimal production at intermediate NaCl concentrations, with the concentration of fish peptone significantly influenced by NaCl concentration in enhancing carotenoid production (Fig. S1a). For KCl, carotenoid production tends to increase with higher concentrations (Fig. S1b, S1d, and S1f). Regarding incubation time, the intermediate levels of NaCl and fish peptone resulted in the maximum carotenoid production, with a decline in production at both higher and lower levels (Fig. S1c and S1e).

Based on these results, the optimized fermentation medium was composed of (g/L): fish peptone 8.0, NaCl 103.2, and KCl 16.2, with an incubation period of 74.4 h. Under these optimized conditions, the predicted maximum carotenoid production was 1.59 mg/L. Experimental validation of these conditions yielded an average carotenoid production of 1.58 mg/L, closely aligning with the model’s predictions. This result validated the accuracy and effectiveness of the optimization model, confirming that the optimized medium is preferable for carotenoid production by “*Hfx. marinum*” MBLA0078. The result demonstrates that CCD is effective for optimizing medium composition and culture conditions when multiple variables influence carotenoid production. Overall, the carotenoid production under DBCM2R optimal conditions (1.58 mg/L) increased by 9.5-fold and 3.2-fold compared to the basal DBCM2 (0.167 mg/L) and DBCM2O (0.501 mg/L) media, respectively, in the flask culture system.

### Scale up production and fed-batch fermentation

**Fig. 2:**
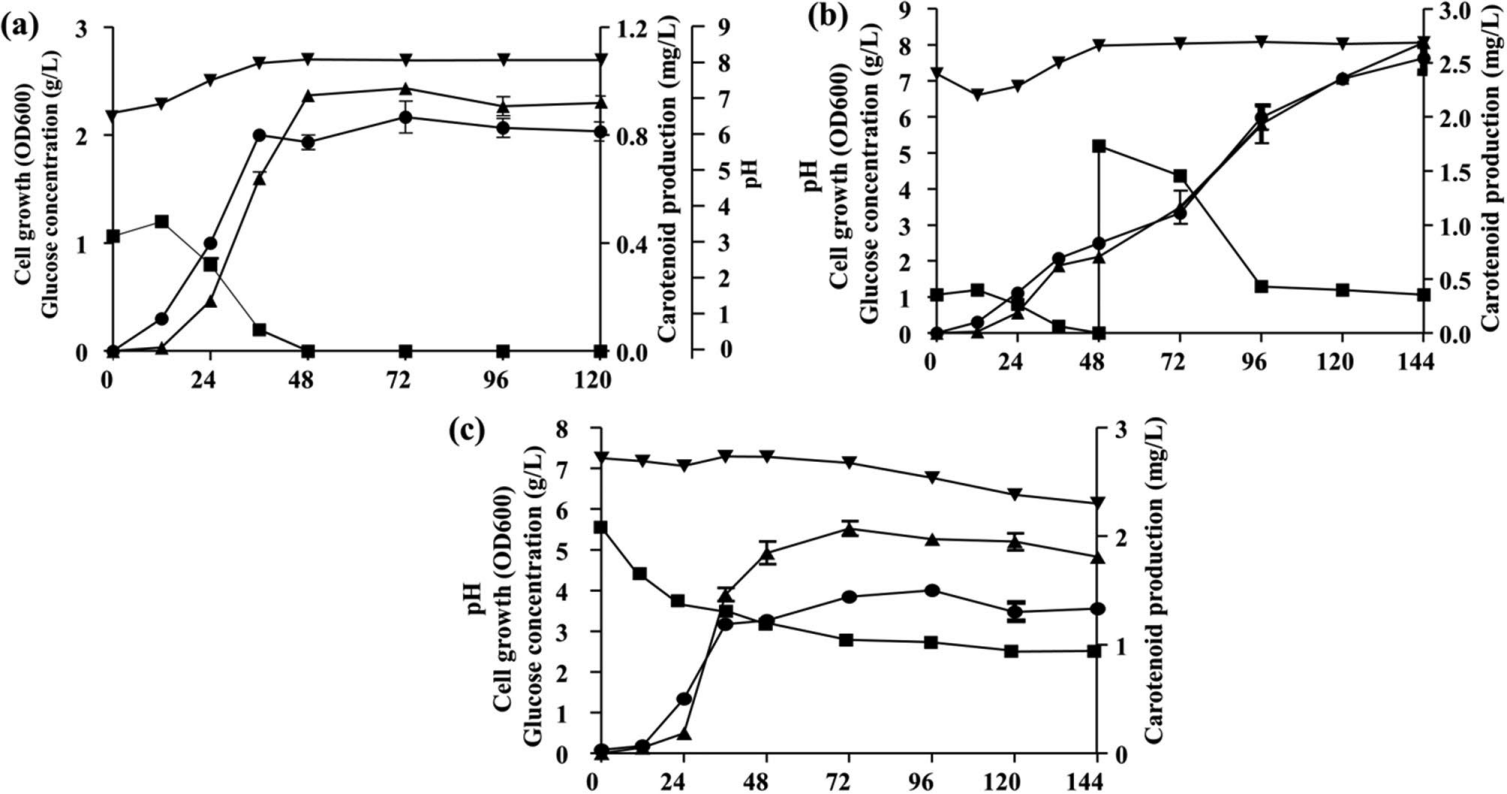
Cho et al. Time-course profiles of the fermentation system: (**a**) in DBCM2O medium; (**b**) in DBCM2O-based fed-batch fermentation with glucose feeding; and (**c**) in DBCM2R medium. Fermentation was conducted at an agitation speed of 500 rpm, with an aeration rate of 1 vvm, at a temperature of 37 °C. Each symbol means cell growth (circle), carotenoids production (triangle), glucose consumption (square), and pH (inverted triangle)

To evaluate the feasibility of OFAT and statistically optimized fermentation media for carotenoid production at a larger scale, “*Hfx. marinum*” MBLA0078 was cultivated in a 7-L laboratory fermenter with a working volume of 4.2 L. In both cases of optimized media, carotenoid accumulation began during the initial exponential phase and continued to increase until the culture reached the stationary phase. In the DMBC2O condition, glucose consumption was closely linked to cell growth and subsequent carotenoid accumulation. A maximum carotenoid production of 0.954 mg/L was achieved within 3 days under optimized conditions (Fig. 2a), with glucose being completely consumed within 48 h. To further enhance carotenoid production, glucose was replenished at 48 h post-consumption to a concentration of 5 g/L. Glucose consumption increased over 48 h, then gradually declined, with no further glucose reduction or carotenoid production observed after 144 h. This glucose feeding strategy resulted in a carotenoid production of 2.80 mg/L (Fig. 2b).

Under the DBCM2R condition, a maximum carotenoid production of 2.16 mg/L was achieved in 3 days, utilizing the optimized medium and fermentation conditions (Fig. 2c). This represents a 2.3-fold increase in carotenoid production compared to the DBCM2O condition (0.954 mg/L) in the laboratory fermenter system. Furthermore, the optimal DBCM2R condition demonstrated a productivity of 0.72 mg/L/day, 1.5 times higher than that achieved with the fed-batch fermentation under DBCM2O conditions (0.47 mg/L/days). These results demonstrate that RSM is a reliable approach for developing predictive models, optimizing culture conditions, and analyzing interaction effects in preparation for industrial carotenoid production. However, the glucose consumption rate was observed to be lower under DBCM2R conditions (Fig. 2c), likely due to the higher nitrogen content in the DBCM2R medium (8.0 g/L fish peptone, 5.5 g/L glucose) compared to the DBCM2O medium (1.0 g/L fish peptone, 1.0 g/L glucose). This suggests that MBLA0078 predominantly relies on organic nitrogen sources in media with high nitrogen content. Previous studies have reported that a lower carbon-to-nitrogen (C/N) ratio in the medium enhances carotenoid production in *Hfx. mediterranei* [40]. These findings emphasize the need for further exploration of haloarchaeal metabolism, particularly as influenced by the C/N ratio. The kinetics of cell growth and stoichiometric analysis were presented in relation to variations in each optimal production medium (Table 6). The values were determined based on the optimal time for carotenoid production under each set of fermentation conditions.

Several studies have reported carotenoid production under various conditions using different halophilic archaea strains (Table 7). Montero-Lobato et al. (2018) optimized carotenoid production in *Hfx. mediterranei* by considering factors such as temperature, pH, and salinity using a central CCD approach. This optimization led to total carotenoid production of 3.74 mg/L over a period of 6 days. Chen et al. (2015) optimized pigment production in *Hfx. mediterranei* by adjusting the conductivity of saline media with extruded rice bran and starch, yielding a maximum of 556 mg/L of carotenoid production. In addition, a carotenoid yield of 0.604 A_494nm_/mL was obtained using a two-stage fermentation system that employed salt stress, as reported by Fang et al. (2010). In “*Hfx. marinum*”, carotenoid production was observed to affect as NaCl concentration (Table S1 − 2), a finding consistent with previous studies. This suggests that NaCl concentration plays a critical role in carotenoid biosynthesis in haloarchaea. In another case, exposure of *Hfx. mediterranei* cells to oxidative stress induced by H_2_O_2_ during the mid-exponential phase led to a concentration-dependent increase in carotenoid production, with yields ranging from 0.55 to 2.13 mg/L (Giani and Martínez-Espinosa 2020). For other haloarchaea strains, CCD-based optimization using *Halorubrum* sp. TBZ126 showed 11.71 mg/L of carotenoid production over 9 days, with adjustments made to temperature, pH, and salinity (Hamidi et al. 2014). Additionally, Hwang et al. (2024) applied a combination of the OFAT-based CCD optimization to “*Halorubrum ruber*”. This approach, which considered factors such as yeast extract, pH, NaCl concentration, inoculum volume, and incubation time, resulted in a carotenoid yield of 1.966 mg/L over 4 days. In another case, an open fermentation system using an unsterile medium with *Halorubrum* sp. HRM-150 enhanced carotenoid production, proving to be cost-effective by eliminating the need for sterilization (Ma et al. 2023).

**Table 7:** Comparison of carotenoid production, production coefficient, and productivity from halophilic archaea strains

Strains	Carotenoid production (mg/L)	Productivity (mg/L/day)	Product yield coefficient (µg/g) ^a^	Reference
“*Haloferax marinum*” MBLA0078	2.16	0.72	977.38	In this study
	2.80	0.47	640.73	
*Haloferax* sp. BKW301	-	-	1589	Biswas et al. (2017)
*Haloferax mediterranei* ATCC 33,500	0.604 A_494nm_/mL	-	-	Fang et al. (2010)
	556	158.857	-	Chen et al. (2015)
	3.74	0.62	2351	Montero-Lobato et al. (2018)
	2.13	-	-	Giani and Martínez-Espinosa (2020)
*Halorubrum* sp. TBZ126	11.71	1.3	-	Hamidi et al. 2014
*Halorubrum* sp. HRM-150	-	-	231	Ma et al. (2023)
“*Halorubrum ruber*” MBLA0099	1.996	0.492	330.23	Hwang et al. (2024)
*Halobacterium salinarium* ATCC 33,171	-	-	45	Mandelli et al. (2012)
*Halobacterium salinarium* R1	-	-	968.7	Kholany et al. (2024)
*Halobacterium halobium* M8	7.63	1.27	-	Abbes et al. (2013)
*Haloarcula japonica* TR-1	-	-	335	Yatsunami et al. (2014)
*Haloarcula* sp. A15	0.734	0.12	-	Shahbazi et al. (2023)
*Halorhabdus utahensis* DSM-12,940	2.428	0.125	550.6	Serino et al. (2023)
*Halococcus morrhuae* ATCC 17,082	-	-	89	Mandelli et al. (2012)
*Haloterrigena turkmenica* DSM-5511	-	-	74.5	Squillaci et al. (2017)
*Haloterrigena thermotolerans* K15	-	-	21.43	Kesbiç and Gültepe (2022)
*Haloterrigena salina* SGH1	-	-	437.5	Flores et al. (2020)

^a^ Product yield coefficient (carotenoid production µg / Biomass g)

In this study, the key factors influencing carotenoid production in “*Hfx. marinum*” were determined through various optimization methods, which also helped uncover the interactions between these factors. This approach enabled a comprehensive understanding of how different variables contribute to enhancing carotenoid production, similar to previous studies that have explored optimization strategies in other haloarchaea species. Although the carotenoid production of “*Hfx. marinum*” MBLA0078 under optimized conditions is lower than some previously reported results, it showed higher productivity (0.72 mg/L/day) than *Hfx. mediterranei* ATCC33550 (0.62 mg/L/day) using RSM as the optimization method (Montero-Lobato et al. 2018). This suggests that the combined OFAT and RSM approaches, coupled with scale-up fermentation, can effectively be employed for industrial-scale carotenoid production using *Haloferax* strains, leading to enhanced productivity through optimized processes.

Nowadays, increased health consciousness is expected to drive greater demand for natural products (Prasath et al. 2024). The pursuit of more sustainable food sources has led to a growing interest in utilizing microbes as hosts for producing acellular products, such as food ingredients (Banovic and Grunert 2023). Genetic engineering and various fermentation methods including traditional, biomass, and precision fermentation have garnered global attention for their pivotal roles in advancing next-generation food ingredients and products (Augustin et al. 2023; Zhang et al. 2024). This approach involved evaluating the performance of biosynthetic genes and analyzing crucial metabolic intermediates to identify flux bottlenecks in the pathway (Li et al. 2021). Additionally, sustainable and cost-effective feedstocks to replace sugar in fermentation processes are under exploration. By-products such as corn steep liquor, rice bran and starch, and crude glycerol are being evaluated as viable, low-cost alternatives to traditional media in precision fermentation, including microbial carotenoid production (Cutzu et al. 2013; Chen et al. 2015; Fallahi et al. 2023). Furthermore, various methods are being developed to achieve precise fermentation optimization, with RSM widely employed to estimate the relationship between decision variables and response variables (Zhang et al. 2024). However, it may be limited in accurately predicting complex response changes. Artificial neural networks (ANNs) have been increasingly utilized to model and control dynamic biological processes, offering new possibilities for intelligent microbial fermentation (Hosen et al. 2011). By combining RSM with ANNs, these limitations can be overcome, offering a robust approach to process optimization (Zhang et al. 2024). This integration enhances the precision and efficiency of optimization efforts. Therefore, different optimization strategies can be integrated into haloarchaea cultures to achieve higher targeted carotenoid productivity. Further optimization experiments aimed at increasing the carotenoid production yield are anticipated to make its production more cost-effective and sustainable.

## Conclusions

Through the application of OFAT and RSM design, combined with scaled-up fermentation, strain MBLA0078 achieved carotenoid production of 0.954 mg/L and 2.16 mg/L, respectively. In fed-batch fermentation using DBCM2O medium, the carotenoid production increased to 2.80 mg/L. RSM design proved to be more effective than OFAT for improving productivity, as it allowed for the evaluation and optimization of multiple influencing factors simultaneously. These findings indicate that conventional OFAT and RSM approaches can significantly enhance carotenoid production by optimizing simple media compositions and culture conditions. This strategy provides valuable insights for advancing the large-scale production of C_50_ carotenoids, which have promising applications in the food, cosmetic, and pharmaceutical industries.

## Electronic Supplementary Material

Below is the link to the electronic supplementary material.

Supplementary Material 1

## Data Availability

All data and materials are available in this article and will be given access on the journal website.
