# Supplementary material for: Optimized production of bacterioruberin from “Haloferax marinum” using one-factor-at-a-time and central composite design approaches
Source: Bioresour Bioprocess. 2024 Dec 19;11(1):111. doi: 10.1186/s40643-024-00834-9 (PMC11659531; doi:10.1186/s40643-024-00834-9)
Supplement: Supplementary file 1 — Supplementary Material 1 [file 40643_2024_834_MOESM1_ESM.docx]

Supporting information for

**Optimized production of bacterioruberin from “*Haloferax marinum*” using one-factor-at-a-time and central composite design approaches**

Eui-Sang Cho^1,2†^, Chi Young Hwang^2†^ and Myung-Ji Seo^2,3,4^*

^1^BioTechnology Institute, University of Minnesota, St. Paul, MN, 55108, USA

^2^Department of Bioengineering and Nano-Bioengineering, Incheon National University, Incheon 22012, Republic of Korea

^3^Division of Bioengineering, Incheon National University, Incheon 22012, Republic of Korea

^4^Research Center for Bio Materials & Process Development, Incheon National University, Incheon 22012, Republic of Korea

^†^Eui-Sang Cho and Chi Young Hwang contributed equally to this work.

***Correspondence:**

Myung-Ji Seo

mjseo@inu.ac.kr

| Source | DF | Sum of squares | Mean square | F-value | P-value Prob>F |
| --- | --- | --- | --- | --- | --- |
| Model | 11 | 2.87 | 0.26 | 62.04 | 0.00 |
| X_1_ | 1 | 0.02 | 0.02 | 5.16 | 0.11 |
| **X_2_ (Fish peptone)** | 1 | 0.13 | 0.13 | 30.00 | 0.01^*^ |
| X_3_ | 1 | 0.03 | 0.03 | 6.90 | 0.08 |
| **X_4_ (NaCl)** | 1 | 0.84 | 0.84 | 199.25 | 0.00^*^ |
| X_5_ | 1 | 0.04 | 0.04 | 8.90 | 0.06 |
| X_6_ | 1 | 0.02 | 0.02 | 3.67 | 0.15 |
| **X_7_ (KCl)** | 1 | 0.16 | 0.16 | 37.22 | 0.01^*^ |
| X_8_ | 1 | 0.00 | 0.00 | 0.87 | 0.42 |
| X_9_ | 1 | 0.00 | 0.00 | 0.34 | 0.60 |
| **X_10_ (Incubation time)** | 1 | 0.31 | 0.31 | 73.86 | 0.00^*^ |
| Residual | 3 | 0.01 | 0.00 |  |  |
| Lack of fit | 1 | 0.00 | 0.00 | 0.63 | 0.51 |
| Pure error | 2 | 0.01 | 0.00 |  |  |
| Cor total | 14 | 2.88 |  |  |  |

**Table S1** ANOVA for the experimental results generated by Plackett-Burman design model

R^2^=0.996; Adj R^2^=0.980

* Model terms are significant

**Table S2** ANOVA of variance for the experimental results generated by central composite design model

| Source | DF | Sum of squares | Mean square | *F*-value | *P*-value Prob>F |
| --- | --- | --- | --- | --- | --- |
| Model | 14 | 3.55 | 0.25 | 17.50 | 0.00^*^ |
| A | 1 | 0.45 | 0.45 | 31.17 | 0.00^*^ |
| B | 1 | 0.51 | 0.51 | 35.15 | 0.00^*^ |
| C | 1 | 0.38 | 0.38 | 26.39 | 0.00^*^ |
| D | 1 | 0.01 | 0.01 | 0.37 | 0.55 |
| A^2^ | 1 | 0.28 | 0.28 | 19.06 | 0.00^*^ |
| B^2^ | 1 | 1.26 | 1.26 | 86.76 | 0.00^*^ |
| C^2^ | 1 | 0.16 | 0.16 | 11.31 | 0.00^*^ |
| D^2^ | 1 | 0.85 | 0.85 | 58.70 | 0.00^*^ |
| A*B | 1 | 0.17 | 0.17 | 11.62 | 0.00^*^ |
| A*C | 1 | 0.03 | 0.03 | 2.23 | 0.16 |
| A*D | 1 | 0.01 | 0.01 | 0.53 | 0.48 |
| B*C | 1 | 0.04 | 0.04 | 3.02 | 0.10 |
| B*D | 1 | 0.01 | 0.01 | 0.59 | 0.45 |
| C*D | 1 | 0.00 | 0.00 | 0.04 | 0.85 |
| Residual | 15 | 0.22 | 0.01 |  |  |
| Lack of fit | 10 | 0.19 | 0.02 | 4.06 | 0.07 |
| Pure error | 5 | 0.02 | 0.00 |  |  |
| Cor total | 29 |  |  |  |  |

R^2^=0.942; Adj R^2^=0.889

* Model terms are significant

**
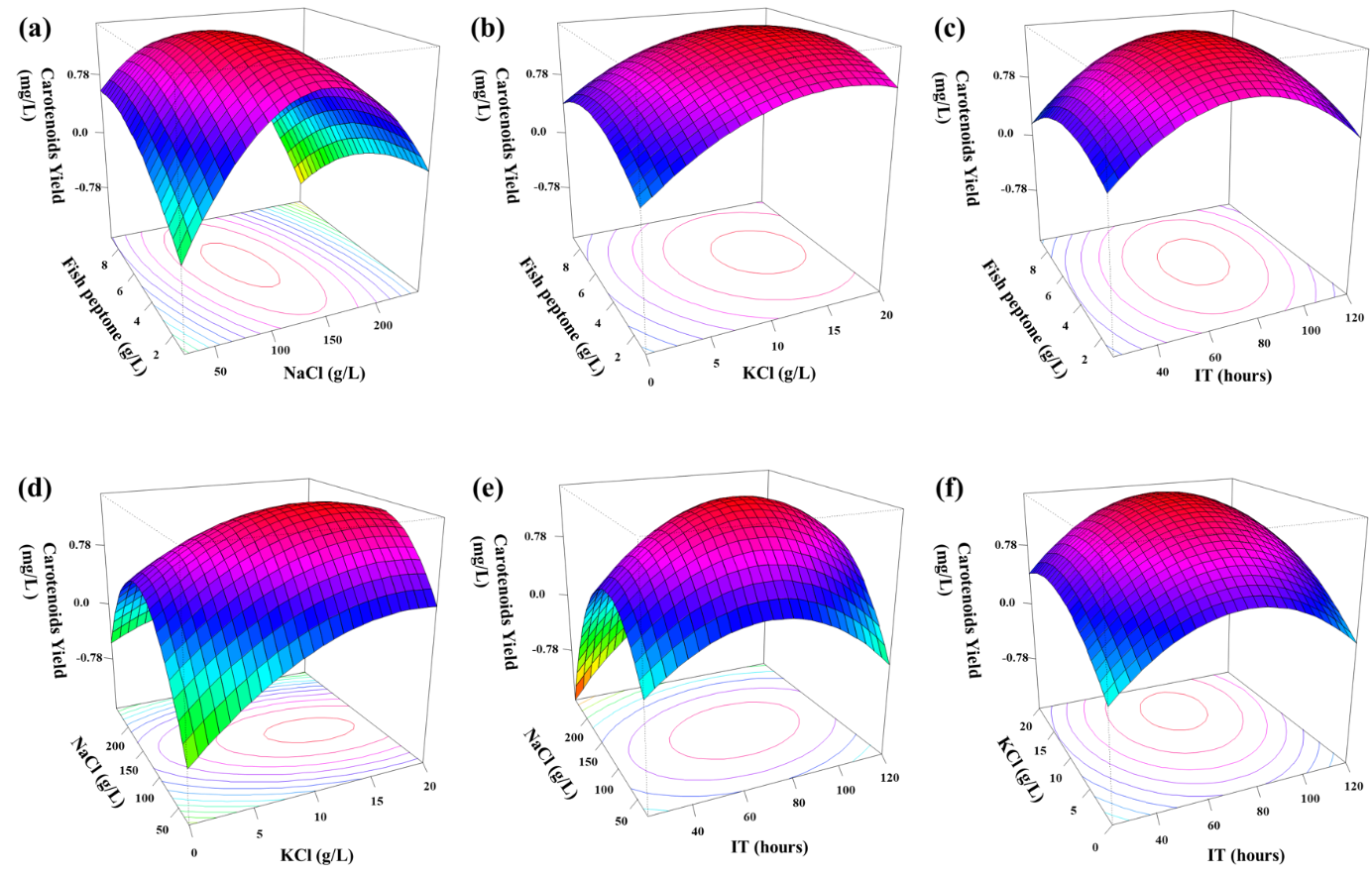
**

**Fig. S1.** Response surface 3D contour plots of carotenoid production from “*Hfx. marinum*” MBLA0078; changing components are (a) Fish peptone and NaCl, (b) Fish peptone and KCl, (c) Fish peptone and IT, (d) NaCl and KCl, (e) NaCl and IT and (f) KCl and IT. Other variables were kept constant
